# Lactate kinetics in ICU patients using a bolus of ^13^C-labeled lactate

**DOI:** 10.1186/s13054-020-2753-6

**Published:** 2020-02-10

**Authors:** Jonathan Grip, Tobias Falkenström, Panuwat Promsin, Jan Wernerman, Åke Norberg, Olav Rooyackers

**Affiliations:** 1 Clinical Science Intervention and Technology (CLINTEC), Department of Anesthesiology and Intensive Care, Karolinska Inititutet, Huddinge, Sweden; 2 Department of Perioperative Medicine and Intensive Care, Karolinska University Hospital, Huddinge, Sweden; 3 Division of Critical Care, Department of Medicine, Faculty of Medicine Siriraj Hospital, Mahidol University, Bangkok, Thailand

**Keywords:** Lactate, Lactate kinetics, Labeled lactate, Stable isotope, ICU, Method validation

## Abstract

**Background:**

Plasma lactate concentrations and their trends over time are used for clinical prognosis, and to guide treatment, in critically ill patients. Although heavily relied upon for clinical decision-making, lactate kinetics of these patients is sparsely studied.

**Aim:**

To establish and validate a feasible method to study lactate kinetics in critically ill patients.

**Methods:**

Healthy volunteers (*n* = 6) received a bolus dose of ^13^C-labeled lactate (20 μmol/kg body weight), and 43 blood samples were drawn over 2 h to determine the decay in labeled lactate. Data was analyzed using non-compartmental modeling calculating rates of appearance (*R*_a_) and clearance of lactate. The area under the curve (AUC) was calculated using a linear-up log-down trapezoidal approach with extrapolation beyond 120 min using the terminal slope to obtain the whole AUC. After evaluation, the same protocol was used in an unselected group of critically ill patients (*n* = 10).

**Results:**

*R*_a_ for healthy volunteers and ICU patients were 12.8 ± 3.9 vs 22.7 ± 11.1 μmol/kg/min and metabolic clearance 1.56 ± 0.39 vs 1.12 ± 0.43 L/min, respectively. ICU patients with normal lactate concentrations showed kinetics very similar to healthy volunteers. Simulations showed that reducing the number of samples from 43 to 14 gave the same results. Our protocol yielded results on lactate kinetics very similar to previously published data using other techniques.

**Conclusion:**

This simple and user-friendly protocol using an isotopically labeled bolus dose of lactate was accurate and feasible for studying lactate kinetics in critically ill ICU patients.

**Trial registration:**

ANZCTR, ACTRN12617000626369, registered 8 March 2017. https://anzctr.org.au/Trial/Registration/TrialReview.aspx?id=372507&isReview=true

## Background

The correlation between elevated plasma lactate concentrations and in-hospital mortality is firmly established in both intensive care and emergency department settings. A failure to normalize an initially elevated lactate concentration is an even stronger predictor of an unfavorable outcome [1–5]. Lactate is therefore used as a triage tool [6–8] and is recommended to be measured within 1 h in suspected sepsis [9]. These relationships are most thoroughly studied in septic patients, but similar correlations are shown in other conditions as well [10].

Although heavily relied upon for clinical decision-making, most knowledge on lactate in critical illness comes from retrospective and prospective observational clinical data. Reports on lactate metabolism and kinetics in ICU patients are sparse. This may be because of a common misconception that lactate is simply the end product of anaerobic metabolism during hypoxia. However, lactate metabolism is far more complex, and plasma lactate concentrations may be altered by e.g. metabolic stress through β-stimulation [11–13] or pharmacological substances [14, 15]. Lactate can also serve as an energy substrate in various organs, such as the heart and the brain, and is a precursor for gluconeogenesis in the liver and kidney [16–19]. Lactate plays a vital role as an energy substrate in septic rats, as animals depleted from endogenous lactate production quickly die, but show improved survival when substitution with exogenous lactate is given [20]. An elevated lactate could potentially arise from either an increase in lactate production or an impairment in lactate utilization (or clearance). Although most data suggest that an increase in lactate production is present [21], there are also reports of an additional impairment in utilization in some subjects from both animal models [22] and septic patients [23] and that this impairment correlates with worsened outcome [22, 24].

To deepen the understanding of lactate metabolism, which could help to improve treatment and avoid potential pitfalls, it is important to investigate lactate kinetics, production as well as clearance, in a variety of patient populations. However, the most frequently used method, with a primed continuous infusion of labeled lactate, will be problematic to use in acutely ill unstable patients. Also, performing extensive sampling in severely ill patients comes with practical and ethical considerations. To facilitate future clinical scientific studies of lactate kinetics, methods with high accuracy, and minimal risk of harm and interference of ongoing care, are needed. We therefore performed a study aiming to develop a relatively simple and feasible protocol for the ICU setting, using a bolus dose of ^13^C-labeled lactate to study lactate kinetics. To achieve this, we initially studied healthy volunteers and subsequently applied the same protocol to ten critically ill patients.

## Methods

### Patient population and ethical considerations

Six healthy volunteers were recruited after oral and written informed consent and screened for past and present illness. ICU patients were screened for eligibility and recruited after informed consent (or from next of kin when appropriate). The study was approved by the regional ethics committee (Stockholm, Sweden, no. 2016/722-31/1), and amendments were approved after the first part (volunteers) was completed. The study was registered at ANZCTR (ACTRN12617000626369).

### Experimental setup

In part I, volunteers were studied after an overnight fast. A peripheral venous catheter and an arterial catheter were inserted after the application of local anesthesia. In part II, all ICU patients had both venous and arterial catheters, as part of ongoing care, prior to enrollment. ICU patients were studied with ongoing nutrition according to the local protocol. For the ICU patients, their ideal weight was assessed as:

$$ \mathrm{Ideal}\ \mathrm{weight}=\mathrm{Height}\ \left(\mathrm{cm}\right)-100 $$

The body weight used for each ICU subject was:

$$ \mathrm{Body}\ \mathrm{weight}=\mathrm{Ideal}\ \mathrm{weight}+\frac{\left(\mathrm{measured}\ \mathrm{weight}-\mathrm{ideal}\ \mathrm{weight}\right)}{3} $$

Measured weight was used for the healthy volunteers.

Baseline samples were drawn, and a ^13^C-labeled lactate containing solution was administered intravenously over 20 s (20 μmol of sodium-lactate/kg body weight, diluted with isotonic NaCl, to a final volume of 20 mL). The amount of lactate given was based on the rate of appearance (*R*_a_) for lactate from our previous study [25], the lactate pool size, and our experience from a bolus approach with labeled glutamine [26]. After baseline samples were drawn, administration of lactate started at *t* = 0 and a total of 42 arterial blood samples were drawn in the following 2 h. Sampling was performed at *t* = 2 min and then every 2 min for the first hour and every 5 min in the following hour. At each sampling, blood was drawn in an EDTA tube, centrifuged at 2000*g* for 10 min, plasma extracted, and immediately frozen and kept at − 80 °C until analysis. During the first 16 min, a total of five additional arterial blood samples were taken and immediately analyzed on a point-of-care blood gas analyzer for plasma lactate concentration. In total, approximately 100 mL of blood was sampled from each subject.

### Laboratory analysis and specifications

1-^13^C-labeled sodium-lactate (Cambridge Isotope Laboratory, Tewksbury, MA, USA) for i.v. administrations was prepared by a licensed pharmacy (APL, Stockholm, Sweden) before use. Arterial blood samples were analyzed for plasma lactate concentrations on a blood gas analyzer (ABL 800 flex, Radiometer Medical Aps, Copenhagen, Denmark). Frozen plasma samples were defrosted and analyzed by gas chromatography-mass spectroscopy (GS-MS) (Inert XL MSD. 5975C, Agilent Technologies, Santa Clara, CA, USA) as previously described [25] for the ^13^C-lactate enrichment and expressed as molar percent excess (MPE).

### Statistics and mathematical modeling

Data was analyzed using Excel (2016, Microsoft Software, Redmond, WA, USA) and Prism (7.02, GraphPad Software, La Jolla, CA, USA).

For each test subject, a decay curve was obtained by plotting enrichment data against time. Non-compartmental analysis was used, where linear elimination from the central compartment was assumed (for details, see Additional file 1). As enrichment did not reach base line within the 2 h, data were transformed to the logarithmic domain and the terminal slope (λz) was determined by extrapolation (from *t* = 70 min). As the first sample was drawn after 2 min and the bolus given over 20 s, we extrapolated the “early” phase (points 2, 4, 6 min) back to *t* = 0.5 min in the log domain, and the corresponding MPE was calculated. A straight line was drawn from zero to this point. These boundaries were then used to calculate the area under the curve (AUC) using a linear-up log-down trapezoidal method according to:

$$ \mathrm{AUC}=\left({t}_2-{t}_1\right)\times \frac{\left({E}_1-{E}_2\right)}{\left(\ln {E}_1-\ln {E}_2\right)} $$

Rate of appearance (*R*_a_; μmol/kg/min) was calculated as:

$$ {R}_{\mathrm{a}}=\frac{\mathrm{Dose}\ \left[{}^{13}\mathrm{CLactate}\right]}{\mathrm{AUC}}\times 100 $$

where Dose [^13^CLactate] refers to the amount of tracer given per kg body weight. *R*_a_ is the amount or lactate that appears into the plasma, and when no exogenous lactate is given, this represents de novo tissue synthesized lactate that is released into the plasma. Clearance (L/min) was calculated as:

$$ \mathrm{Clearance}=\frac{R_a\times \mathrm{body}\ \mathrm{weight}}{\left[\mathrm{Lactate}\right]} $$

Three patients had ongoing continuous renal replacement treatment (CRRT) during the study period. Loss of lactate through this route was assessed by collecting dialysate during the study period and measure volume and lactate concentration of the dialysate. CRRT clearance of lactate was calculated as the excreted amount per time divided by plasma concentration. Finally, CRRT clearance was subtracted from total clearance (given by the above formula) to get a metabolic clearance comparable to the other subjects.

The non-compartmental pharmacokinetic analysis was performed in accordance with reference text books [27]. Further details and equations are presented in Additional file 1.

Simulations to determine the accuracy with a lower number of samples were performed, using the following time points: baseline, 2, 4, 6, 8, 16, 30, 60, 70, 80, 90, 100, 110, and 120 min.

The healthy volunteers and the ICU patients were compared using an unpaired two-sided *t* test with Welch’s correction (not assuming the same standard deviation in both groups) or Mann-Whitney test, respectively, for normal and non-normal distributed data according to Shapiro-Wilks normality test. Results are expressed as mean ± SD or median (range).

## Results

Baseline characteristics for the volunteers and ICU patients are presented in Tables 1 and 2. The volunteers were younger, had a similar body weight (83 vs 79 kg), and had a smaller male predominance (67% vs 90%).

**Table 1:** Baseline characteristics of healthy volunteers

Age (years)	Sex	Weight (kg)	Height (cm)	Plasma lactate (mmol/L)
23	M	84	187	0.9
23	M	77	187	0.6
47	F	74	165	0.6
54	F	66	165	0.4
32	M	109	182	0.8
23	M	88	185	0.8

**Table 2:** ICU patients

Age (years)	Sex	Weight (kg)	Height (cm)	ICU diagnosis	Plasma lactate (mmol/L)	Alive at 30 days	CRRT	SAPS 3	SOFA
42	M	87	181	Liver transplantation	2.1	Y	Y	27	9
70	M	78	187	Respiratory insufficiency	1.3	Y	N	56	2
75	M	75	184	Respiratory insufficiency	0.7	Y	N	51	7
35	F	38	167	Intoxication	0.6	Y	N	29	2
61	M	80	185	Bowel ischemia	0.6	Y	N	45	6
50	M	77	176	Liver failure	4.9	N	Y	55	5
73	M	90	178	Sepsis	1.5	Y	N	56	4
75	M	88	175	Fluid overload	0.8	Y	N	66	5
63	M	99	198	Pneumonia	3.5	N	Y	55	9
60	M	76	178	Respiratory insufficiency	4.0	N	N	50	12

*LOS* length of stay, *CRRT* continuous renal replacement therapy during the study period, *SAPS* Simplified Acute Physiology Score (at admission), *SOFA* Sequential Organ Failure Assessment score (at the day of investigation)

In the healthy volunteers, 2 samples (0.8%) were lost due to mishandling; in ICU patients, a total of 13 samples (3%) were lost in two patients due to arterial line malfunction. Missing data were not replaced but handled by the log-down trapezoidal method.

**Fig. 1:**
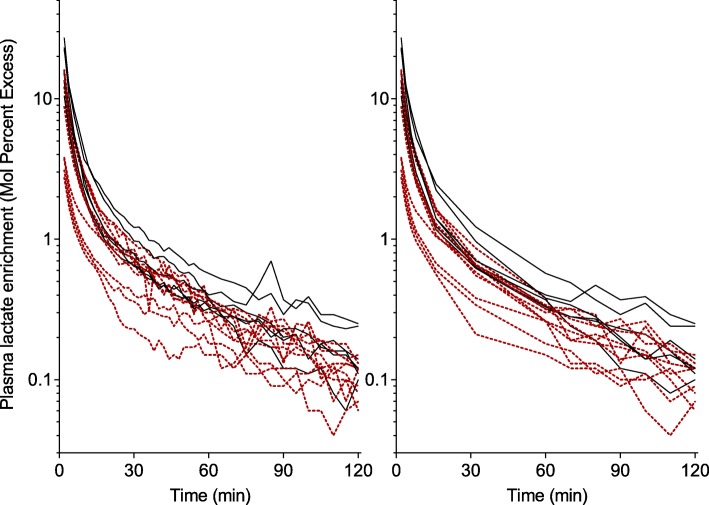
Plasma enrichment of labeled lactate over time in all subjects. Decay curves of plasma lactate enrichments after a bolus dose of ^13^C-labeled lactate in healthy volunteers (filled black lines, *n* = 6) and ICU patients (red dotted lines *n* = 10). The left panel includes all values, 43 samples per subject, and the right panel shows a reduced number, 14 samples per subject

The ^13^C-lactate bolus resulted in decay curves that allowed for calculations of the AUC (Fig. 1 and Additional file 2). Since ^13^C-lactate levels had not returned to baseline at 120 min, the terminal slope was estimated and the whole extrapolated AUC used for kinetic calculations. The extrapolated AUC for the volunteers and the ICU patients were 6.7 and 9.7% of the total AUC, respectively (Table 3). In healthy volunteers, the administration of labeled lactate increased plasma lactate slightly (0.1–0.2 mmol/L), after 2–4 min, but concentrations had returned to baseline at after 8 min in all subjects. In ICU patients, this effect was even less pronounced and all subjects returned to baseline lactate concentrations at *t* = 4 min (Additional file 3: Fig. S1).

**Table 3:** Comparison for lactate kinetic parameters between volunteers and ICU patients by non-compartmental analysis

	Volunteers (*n* = 6)	ICU patients (*n* = 10)	*p*
AUC total [% × min]	172 ± 50	105 ± 43	0.021
AUC_extrap_ [% × min]	11.6 ± 7.8	9.0 ± 4.9	0.474
AUC_extrap_ [% of AUC total]	6.7 ± 4.2	9.7 ± 7.0	0.300
*R*_a_ [μmol/kg/min]	12.8 ± 3.9	22.7 ± 11.1	0.025
*R*_a_ [mmol/min]	1.1 ± 0.5	1.8 ± 0.9	0.067
Metabolic clearance [L/min]	1.56 ± 0.39	1.12 ± 0.43	0.058
Plasma lactate [mmol/L]	0.7 (0.4–0.9)	1.4 (0.6–4.9)	0.065
AUMC total [% × min^2^]	5138 ± 2350	3899 ± 1358	0.263
MRT [min]	25.8 (21.6–51.2)	33.3 (22.8–82.2)	0.076
*V*_ss_ [L]	45.4 ± 14.0	42.0 ± 14.7	0.649
*V*_c_ [L]	9.6 ± 2.5	9.7 ± 3.0	0.937

Values are presented as means ± standard deviation or medians (range) as appropriate according to Shapiro Wilk’s normality test. *p* values refer to unpaired two-sided *t* test with Welch’s correction (not assuming the same standard deviation in both groups) or Mann-Whitney test for nonparametric data. *AUC* area under the curve, *AUC*_*extrap*_ the part of AUC that is beyond the last measurement point, *R*_*a*_ rate of appearance, *AUMC* area under the first moment curve, *MRT* mean residence time, *V*_*ss*_ volume of distribution at steady state, *V*_*c*_ central volume of distribution. Details of the pharmacokinetic non-compartmental modeling are presented in Additional file 1

**Fig. 2:**
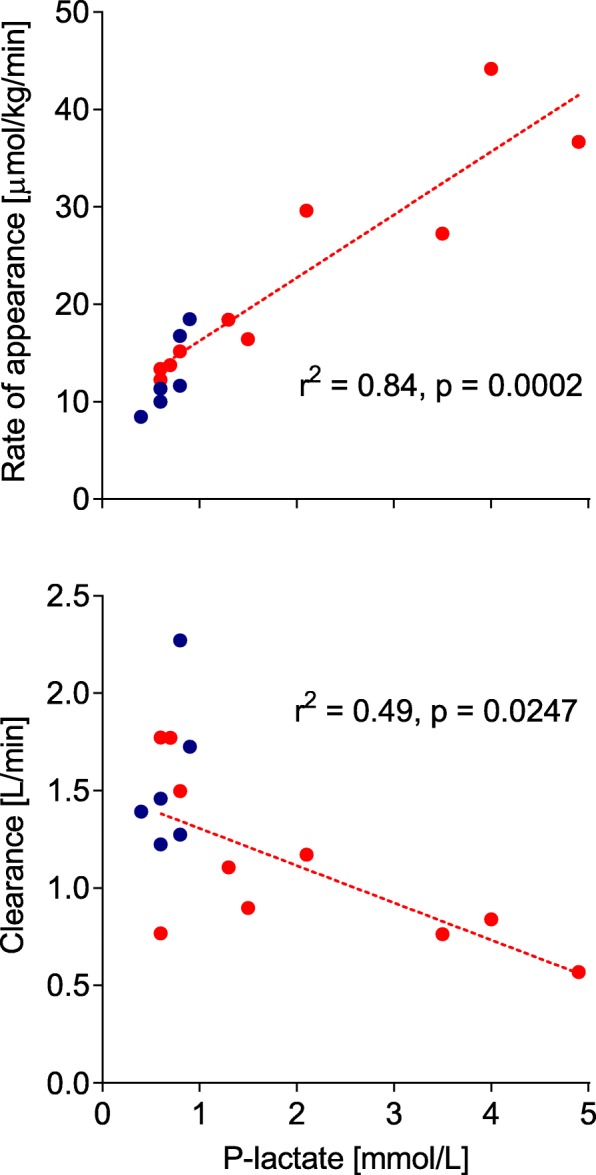
Relationship between plasma concentration and rate of appearance and clearance of lactate. Relation between plasma lactate concentrations and rate of appearance of lactate (upper panel) and metabolic clearance of lactate (bottom panel) as calculated from a bolus dose of ^13^C-labeled lactate in healthy volunteers (blue circles, *n* = 6) and ICU patients (red circles, *n* = 10). The lines of regression and associated statistics apply to the group of ICU patients only

The main findings are presented in Table 3. Plasma lactate concentration and *R*_a_ for healthy volunteers and ICU patients were 0.7 (0.4–0.9) vs 1.4 (0.6–4.9) mmol/L, *p* = 0.065, and 12.8 ± 3.9 vs 22.7 ± 11.1 μmol/kg body weight/min, *p* = 0.025, respectively. Metabolic clearances were 1.56 ± 0.39 vs 1.12 ± 0.43 L/min, *p* = 0.058. For the 3 patients on CRRT, the CRRT clearance contributed with 2.4 to 4.7% of the total lactate clearance. Central volume of distribution and volume of distribution at steady state for labeled lactate were 9.6 ± 2.6 vs 9.7 ± 3.0 L, *p* = 0.9 and 45.4 ± 14.0 vs 42.0 ± 14.7 L, *p* = 0.65. Absolute *R*_a_ was 1.1 ± 0.5 vs 1.8 ± 0.9, mmol/min, *p* = 0.07, for healthy volunteers and ICU patients, respectively. For measurements in the ICU patients, there were statistically significant correlations between plasma concentration and *R*_a_ of lactate, *r*^2^ = 0.84 (*p* < 0.0002), and lactate metabolic clearance, *r*^2^ = 0.48 (*p* = 0.025) (Fig. 2).

When the number of blood samples was reduced to 14, the decay curves were similar to the full protocol using 43 samples (Fig. 1) and the impact on the results of the non-compartmental analysis was negligible (Table 4).

**Table 4:** Comparison between 43 and 14 sample protocols

	*N* = 43	*N* = 14	*p*
AUC_tot_ [% × min]	130 ± 55	132 ± 57	0.054
AUC_extrap_ [% × min]	10.0 ± 6.0	10.3 ± 7.2	0.538
λz 70 [min^−1^]	− 0.015 ± 0.006	− 0.015 ± 0.006	0.748
Ra [μmol/kg/min]	15.8 (8.5–44.2)	15.8 (8.3–44.2)	0.404

Mean ± SD or median (range) for all subjects (healthy volunteers and ICU patients). Differences between protocols are analyzed by paired *t* test or Wilcoxon test for nonparametric data. *AUC*_*tot*_ area under the curve, *AUC*_*extrap*_ the part of AUC that is beyond the last measurement point, *λz 70* terminal slope as calculated from *t* = 70 min, *R*_*a*_ rate of appearance of lactate. Details of the pharmacokinetic non-compartmental modeling are presented in Additional file 1

When ICU patients were dichotomized into groups with normal plasma lactate (≤ 2 mmol/L, *n* = 6, median lactate 0.9 mmol/L) and elevated plasma lactate (> 2 mmol/L, *n* = 4, median lactate 3.8 mmol/L), those with normal plasma concentrations had lactate kinetics more similar to the healthy volunteers as *R*_a_ (both absolute and per body weight) and plasma clearance was almost identical (Table 5).

**Table 5:** Comparison between volunteers and ICU patients with normal and elevated plasma lactate

	Volunteers (*n* = 6)	ICU, normal lactate (*n* = 6)	ICU, elevated lactate (*n* = 4)
P-lactate [mmol/L]	0.7 (0.4–0.9)	0.8 (0.6–1.5)	3.8 (2.1–4.9)
*R*_a_ [μmol/kg/min]	11.5 (8.5–18.5)	14.5 (12.3–18.4)	33.2 (27.3–44.2)
Metabolic clearance [L/min]	1.43 (1.22–2.27)	1.30 (0.77–1.77)	0.80 (0.57–1.17)
*V*_c_ [L]	9.0 (7.0–14.2)	8.4 (5.3–13.5)	10.3 (7.6–14.7)

Normal lactate ≤ 2 mmol/L, elevated lactate > 2 mmol/L. Data are presented as median (range). *R*_*a*_ rate of appearance of lactate, *V*_*c*_ central distribution volume

## Discussion

In this study, we examined a tracer bolus approach to study lactate kinetics in critically ill ICU patients. The feasibility was first demonstrated in healthy volunteers with normal plasma lactate concentrations. The protocol was then repeated in an unselected group of critically ill ICU patients, with normal or elevated plasma lactate concentrations, with comparable feasibility. Simulations showed that the accuracy of the method was unchanged when sampling was decreased from 43 to 14 samples. Therefore, we advocate the use of this technique in clinical studies of lactate kinetics.

The ^13^C-lactate bolus initially increased the lactate concentrations slightly, but the levels returned to baseline within 8 min and are unlikely to affect the measured lactate kinetics over the 120 min. This was expected, as the bolus we gave (20 μmol/kg) was equivalent to roughly twice the rate of appearance per minute in the healthy volunteers. Lowering the bolus would compromise the accuracy of the MPE measurements and the calculation of the AUC. About 10% of the total AUC of the decay curve is after the 120 min sampling period, suggesting that it is important to include the terminal slope in the kinetic calculations. We corrected the clearance for the clearance via loss with CRRT. Although the contribution of the CRRT clearance was less than 5% of the total clearance, it is important to measure this and correct for it. This number may seem small as lactate is a small molecule that is easily dialyzed. However, lactate has a high turnover with high production and clearance (rate of disappearance from plasma) throughout the body and therefore the percentage of the turnover that is removed by dialysis is low.

Continuous infusions of labeled lactate have been used to study lactate metabolism in different populations. We wanted to validate a bolus approach in the ICU settings to stay independent of the underlying assumptions of the continuous infusion approach, such as the appropriate size of the priming dose, and the assumption of a tracer steady state. Both assumptions are problematic in ICU patients, especially in the acute unstable clinical situation when lactate metabolism is most interesting to study. To determine the usefulness and validity of our protocol, the results should be similar to the results obtained with other methods. In resting healthy volunteers (all male and younger), lactate *R*_a_ is 11 ± 2 μmol/kg body weight/min [25], similar to the present group of volunteers. This is also close to the *R*_a_ of 15 and 12 μmol/kg/min that are reported in lean and obese healthy humans [28] and in the healthy volunteers used as controls by Revelly et al. (11.2 ± 2.7 μmol/kg/min) [29], all using different protocols for continuous infusion of labeled lactate. During rest, elite cross-country skiers produce roughly the same amount of lactate, approximately 1.5 mmol/min [30], as volunteers in the present study (1.1 mmol/min). In conclusion, our results agree with previously published results using different methodology in healthy human subjects, supporting the validity of our protocol.

In one of the few studies on lactate metabolism in ICU patients, Revelly et al. [29] used a complex protocol infusing labeled lactate at a rate of 10 μmol/kg/min, which after 2 h was increased to 20 μmol/kg/min for another 2 h. Unfortunately, this protocol may affect lactate metabolism as the infused amount almost matches the endogenous lactate production. This is acknowledged by the authors, whom only report production during the lower infusion rate. They report a *R*_a_ of 26 ± 11 μmol/kg/min in severe sepsis (*n* = 7) and 26 ± 5 μmol/kg/min in cardiogenic shock (*n* = 7).

Levraut et al. assessed lactate kinetics without the use of labeled substrates but by administering 1 mmol/kg sodium-lactate and measuring the subsequent decrease in plasma lactate in hemodynamically stable, normolactemic, and slightly hyperlactemic septic patients (total *n* = 34) [23]. Both the normal and slightly hyperlactemic patients showed similar lactate production rates (20 ± 5 vs 20 ± 4 μmol/kg/min) but differed in clearance (1.0 ± 0.28 vs 0.47 ± 0.10 L/kg/h, *p* < 0.0001). In their subsequent trial, they included septic ICU patients with lactate concentration < 3 mmol/L (*n* = 56) [24]. In this trial, survivors, in comparison to non-survivors, have a higher lactate clearance (0.86 ± 0.32 vs 0.58 ± 0.18 L/kg/h, *p* = 0.016) and a tendency towards a higher lactate production (20 ± 10 vs 15 ± 4 μmol/kg/min, *p* = 0.055), but no difference in plasma lactate concentration. Both of these studies, using the unlabeled lactate, show similar *R*_a_ and clearance rates as the ICU patients in the present study. However, this methodology has limitations as the subject’s lactate concentration actually increases by approximately 1.5 mmol/L, which makes it more difficult to assume that the measurement itself does not affect lactate metabolism.

In both healthy volunteers and ICU patients, the initial volume of distribution (*V*_c_) was 10 L, which may correspond to the body’s visceral high flow compartment. The total volume of distribution (*V*_ss_) was approximately 43 L, which is in the same range as total body water. We find these results plausible since lactate, a small, water-soluble, charged molecule, is likely to behave in the same way as other similar molecules such as ethanol [31].

As one of the aims of this study was to establish a user-friendly protocol, we examined whether reducing the number of blood samples was possible without affecting the results. Simulations showed that decreasing sampling from 43 to 14 samples did not alter kinetic rates (Table 4). This indicates that a reliable protocol is possible with approximately 30 mL of blood sampling.

Within our approach, we made some assumptions. As we did not sample before *t* = 2 min, we have no means of knowing the exact enrichment of labeled lactate before that time point. We chose to extrapolate back to 0.5 min (rather than 0 min) which will give a short time for the substance to mix evenly throughout the blood, since we gave the bolus over the first 20 s. Previously, Avram et al. have shown that concentrations of administered substances show great variability in the first minute, especially in conditions with affected cardiac output [32], which could pose a problem in hemodynamically affected patients. We therefore assume that our approach will give a reasonable average of the levels of enrichment during the first 2 min.

In this study, we made statistical comparisons between the results from the healthy volunteers and the unselected ICU patients (Table 3). We are aware that these two groups do not belong to the same cohort, regarding e.g. background physiology, and are therefore in a strict sense not comparable. The statistical comparison should therefore be seen just as a comparison of the numerical values and be interpreted with care. On the other hand, the correlations of the *R*_a_ and clearance with lactate concentrations in the ICU patients showed a strong correlation between lactate levels and lactate metabolism. Also, when the hyperlactemic ICU patients were excluded, the normolactemic ICU patients and the healthy volunteers had very similar lactate kinetics.

The patients with elevated plasma lactate had a higher *R*_a_ and possibly a lower clearance as compared to normolactemic ICU patients (Table 5). From the correlations (Fig. 2) between lactate concentrations and *R*_a_ and clearance, we can hypothesize that critically ill patients with elevated lactate have high lactate production rates as well as low clearance. Since the study was not designed to investigate these differences, no statistical analysis is presented and we consider the results as hypothesis generating. The main aim of this study was to design a protocol that can be used to study lactate kinetics in various, larger, ICU cohorts. Hopefully, these future studies will allow us to differentiate between patients with different lactate kinetics and thereby improve the care of critically ill patients.

## Conclusion

In conclusion, we present a feasible, reliable, and user-friendly protocol that yields similar results as more complex protocols to elucidate lactate kinetics in healthy volunteers as well as ICU patients. This may be useful for larger studies on lactate metabolism in septic and other patient groups, with the aim to study to what degree high production rates or low clearance rates contribute to hyperlactatemia.

## Supplementary information

**Additional file 1.** Complete documentation of formulas used for mathematical modelling of data.

**Additional file 2.** Plasma lactate enrichment of labeled lactate for healthy volunteers and ICU patients

**Additional file 3.** Individual values for lactate concentrations in plasma for healthy volunteers and ICU patients during experiment.

## Data Availability

The datasets used and analyzed during the current study are available from the corresponding author on reasonable request.
