# Supplementary material for: Lactate kinetics in ICU patients using a bolus of 13C-labeled lactate
Source: Crit Care. 2020 Feb 10;24:46. doi: 10.1186/s13054-020-2753-6 (PMC7011254; doi:10.1186/s13054-020-2753-6)
Supplement: Supplementary file 1 — Additional file 1. Complete documentation of formulas used for mathematical modelling of data. [file 13054_2020_2753_MOESM1_ESM.pdf]

Additional file 1: Supplementary equations for data analysis with illustrations

Non-compartmental analysis (NCA), data analysis

Non-compartmental analysis was performed in accordance with reference text books [1-2]. The only exception was related to the 20 sec infusion time in peripheral vein and sampling in the arterial line. This made us use the time = 30 sec as the presumed time for maximum enrichment in the calculations, to avoid overestimation of the area under the curve (AUC, **Fig S1 a**).

The following parameters were calculated from the enrichment (E, expressed as mol percent excess) versus time plots:

E_0 was calculated by back extrapolation of the first 3 measurements of tracer enrichment at time points 2, 4 and 6 min in the log domain. The slope was also calculated. Then $E_{0.5min}$ was calculated (**Fig S1 a**) as

$$E_{0.5} = e^{(\ln(E_0)+0.5*slope))} \tag{1}$$

The terminal slope (λz) and corresponding intercept were calculated in the log E domain from all measurement time points between 70 and 120 min (**Fig S1 b**).

The calculated last measurement (E_{last}) was calculated by the above parameters as

$$E_{last} = e^{(intercept+\lambda_z*t_{last})} \tag{2}$$

where t_{last} is 120 min (**Fig S1 b**).

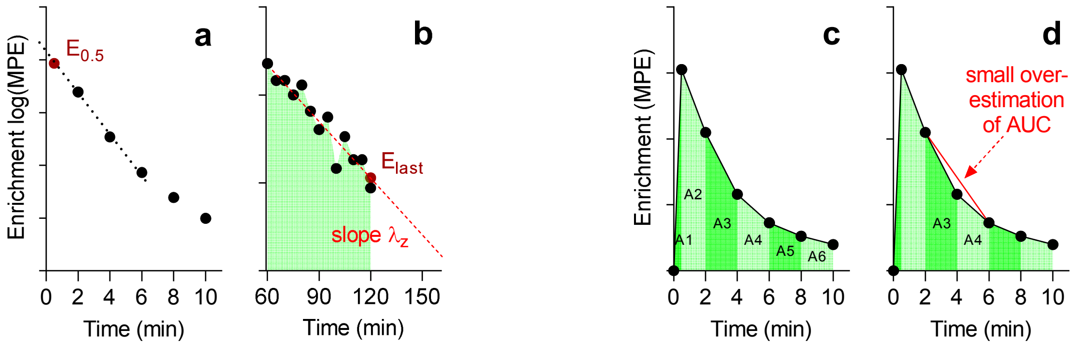

Fig S1. Illustrations of AUC calculations. *Panel **a** demonstrates back-extrapolation in the log domain to enrichment at 0.5 min ($E_{0.5}$), panel **b** extrapolation beyond the last measurement time point to achieve the calculated last enrichment (E_{last}) and the terminal slope (λ_z), again in the log domain. Panel **c** is a presentation of the general AUC concept (no logarithms) and panel **d** demonstrates why a missing value at 4 min will cause a small overestimation of AUC if the trapezoidal rule is used. This can however be greatly eliminated by the log-down method.*

Area under the curve (AUC) was calculated by the lin-up log-down approach as described in the main paper. The general principle of AUC calculations is presented in **Fig S1 c**, where AUC = A1+a2+A3…. When reducing the number of samples there is an overestimation of AUC as demonstrated by the area between the black and red lines in **Fig S1 d**.

The part of AUC beyond the last measurement (AUC_{extrap}) was calculated as

$$AUC_{extrap} = \frac{E_{last}}{\lambda_z} \tag{3}$$

The total area under the curve (AUCtot) is then

$$AUC_{tot} = AUC_{last} + AUC_{extrap} \tag{4}$$

and the fraction of AUC after the last measurement point ($AUC_{extrap\%}$) is

$$AUC_{extrap\%} = 100 * \frac{AUC_{extrap}}{AUC_{tot}} \tag{5}$$

Rate of appearance (Ra) can be calculated as

$$R_a = \frac{tracer\ dose}{AUC_{tot}} \tag{6}$$

Clearance was calculated as

$$Cl = \frac{R_a * body\ weight}{plasma\ lactate} \tag{7}$$

The moment curve was constructed by multiplying E values by time, and the area under the moment curve (AUCM) was calculated by the same principles as the AUC using a linear up log down approach. The part of AUCM beyond the last measurement point was calculated by

$$AUCM_{extrap} = \frac{E_{last} * t_{last}}{\lambda_z} + \frac{E_{last}}{{\lambda_z}^2} \tag{8}$$

The total area under the moment curve ($AUCM_{tot}$) is then

$$AUCM_{tot} = AUCM_{0-last} + AUCM_{extrap} \tag{9}$$

Mean residence time (MRT) was calculated by

$$MRT = \frac{AUCM_{tot}}{AUC_{tot}} \tag{10}$$

The distribution volume at steady state (Vss) is then

$$V_{ss} = MRT * C \tag{11}$$

The volume of the central compartment (Vc) was derived by

$$V_c = \frac{Tracer\ dose}{E_{0.5}} \tag{12}$$

References

1. Pharmacokinetic and Pharmacodynamic Data Analysis: Concepts and Applications, 4th edition, Gabrielsson J and Weiner D, Swedish Pharmaceutical Press, Stockholm, Sweden, 2006

2. Clinical Pharmacokinetics and Pharmacodynamics: Concepts and Applications, 4th edition, Rowland M and Tozer TN, Lippincott Williams and Wilkins, Philadephia, 2010
