# Supplementary material for: Lactate kinetics in ICU patients using a bolus of 13C-labeled lactate
Source: Crit Care. 2020 Feb 10;24:46. doi: 10.1186/s13054-020-2753-6 (PMC7011254; doi:10.1186/s13054-020-2753-6)
Supplement: Supplementary file 2 — Additional file 2. Plasma lactate enrichment of labeled lactate for healthy volunteers and ICU patients [file 13054_2020_2753_MOESM2_ESM.pdf]

Additional file 2

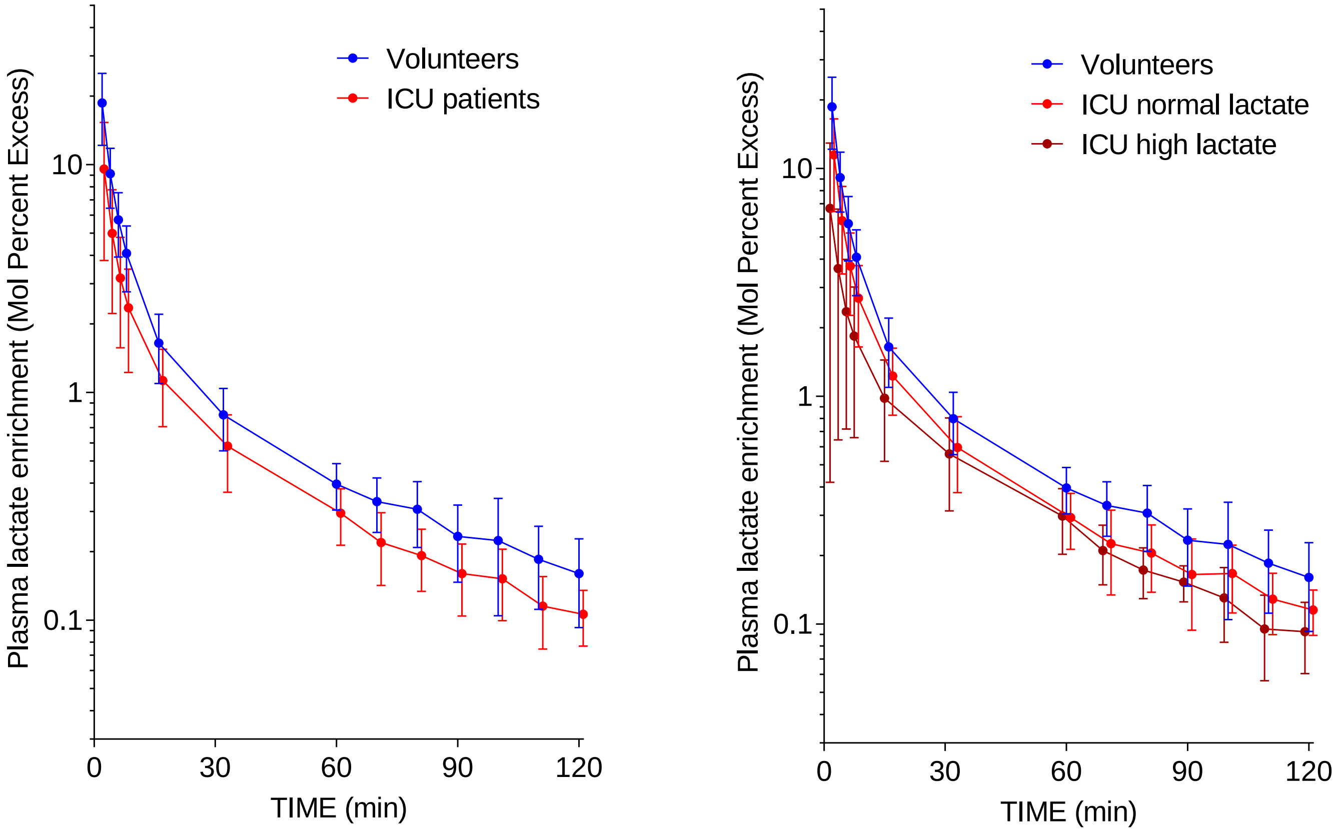

Alternative presentations of plasma lactate enrichment versus time after a bolus injection of ^{13}C-lactate in 6 volunteers and 10 ICU patients.

In the left panel data are presented as mean ± standard deviation for volunteers (blue) and ICU patients (red), respectively. In the right panel the ICU-patients are divided into two groups with high (n=4, dark red) or normal (n=6, red) plasma lactate.
