# Supplementary material for: Lactate kinetics in ICU patients using a bolus of 13C-labeled lactate
Source: Crit Care. 2020 Feb 10;24:46. doi: 10.1186/s13054-020-2753-6 (PMC7011254; doi:10.1186/s13054-020-2753-6)

Additional file 3

Plasma lactate concentrations for healthy volunteers (top, n=6)) and ICU patients (bottom, n=10) after a bolus dose of ^{13}C-labeled lactate during 20 seconds at t=0. In one healthy subject the blood gas analyzer was malfunctioning. Plasma lactate for this subject was reanalyzed from the frozen plasma sample according as previously described [25].

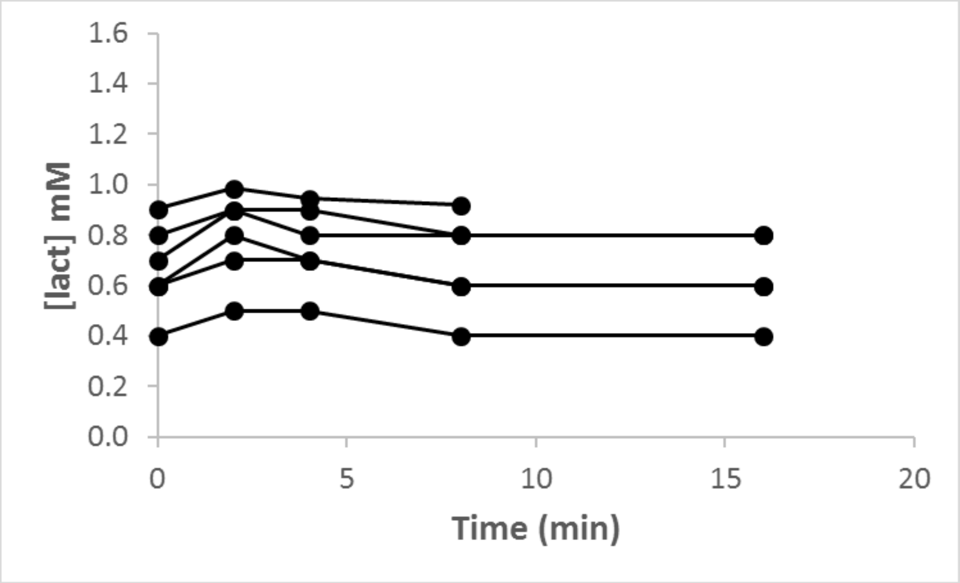

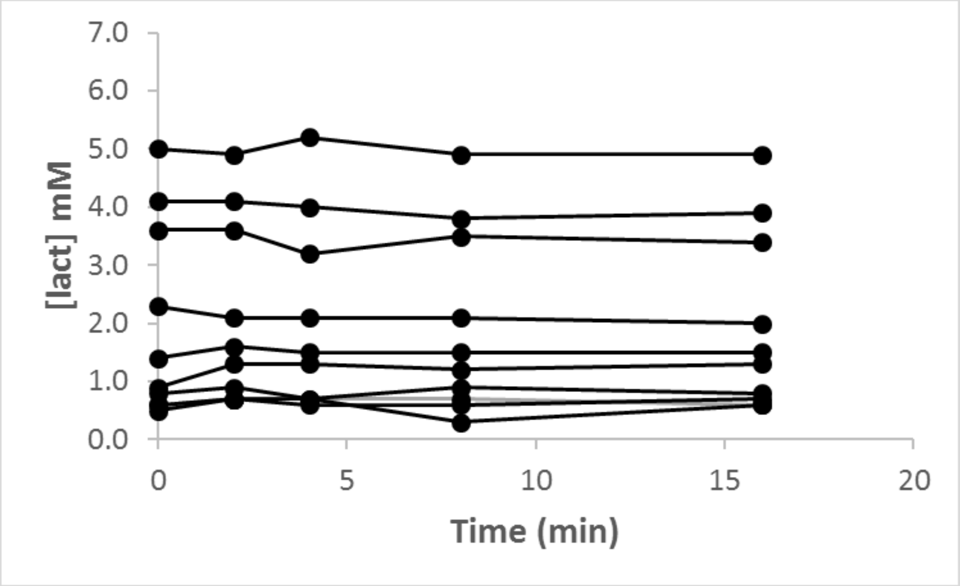

Supplement: Supplementary file 3 — Additional file 3. Individual values for lactate concentrations in plasma for healthy volunteers and ICU patients during experiment. [file 13054_2020_2753_MOESM3_ESM.pdf]
